# The relationship between physical fitness and somatosensory brain responses in adolescents—a pilot study

**DOI:** 10.3389/fspor.2026.1814226

**Published:** 2026-04-24

**Authors:** Aoi Mase, Masako Nakagawa, Manabu Shibasaki, Hiroki Nakata

**Affiliations:** 1 Graduate School of Humanities and Sciences, Nara Women’s University, Nara, Japan; 2 Nara Women’s University Secondary School, Nara, Japan; 3 Faculty of Engineering, Nara Women’s University, Nara, Japan

**Keywords:** EEG, motor skills, physical fitness, SEPs, somatosensory processing

## Abstract

**Introduction:**

This study examined whether physical fitness is associated with somatosensory brain responses during puberty, using somatosensory evoked potentials (SEPs) and anthropometric/physical fitness indices.

**Methods:**

Sixteen boys and twenty girls participated. Height, weight, 50-m sprint, standing long jump, grip strength, sit-ups, sit and reach, side steps, and ball throw were assessed. SEPs were elicited by electrical stimulation of the right median nerve (3 Hz). Data from Fz and C3′ were analyzed. Peak-to-peak amplitudes were measured for N15, P18, and N30 at Fz and for N18, P22, N27, P45, and N60 at C3′. Correlations were tested between physical fitness measures (including total score) and SEP amplitudes.

**Results:**

In boys, the Fz N30 amplitude was correlated with total score (*r* = 0.594, *p* = 0.019), 50-m sprint performance (*r* = −0.560, *p* = 0.024), and sit-ups (*r* = 0.657, *p* = 0.008). In girls, standing long jump distance was negatively correlated with the C3′ N27 amplitude (*r* = −0.585, *p* = 0.007).

**Conclusion:**

Significant associations were observed between specific physical fitness components and somatosensory SEP amplitudes in adolescents, with distinct patterns identified in boys and girls. These findings indicate that somatosensory brain responses are related to physical fitness during puberty.

## 1 Introduction

Physical fitness components, such as running, jumping, and throwing, are closely linked to health in children and form the foundation for participation in sports and daily physical activity (1). The development of physical fitness is influenced by multiple factors, including cardiorespiratory fitness, relative age, and habitual physical activity (2–4). In addition to these functional and behavioral factors, anthropometric characteristics may also modulate physical fitness-related outcomes. For example, Qin et al. (5) reported an inverted U-shaped relationship between body mass index (BMI) and physical fitness in adolescents, suggesting that physical characteristics can interact with fitness outcomes (5). Taken together, previous findings suggest that development of physical fitness may be influenced not only by practice and maturation, but also by physical condition and lifestyle.

Previous studies examining the neural correlates of physical fitness largely focused on cognitive aspects of motor performance, particularly executive function. This function broadly refers to higher-order cognitive processes supporting goal-directed behavior, and is commonly conceptualized as comprising inhibition, working memory, and cognitive flexibility (6, 7). Several studies reported associations between aerobic capacity and executive function in children (8, 9). However, these studies primarily emphasize cognitive processes rather than sensory mechanisms directly involved in motor control.

Somatosensory processing is fundamental for successful motor execution, as it provides continuous feedback necessary for coordination, balance, and movement adjustment (10). Many previous studies investigating the relationship between somatosensory processing and motor abilities focused on athlete populations. For example, Mizuguchi et al. (11) demonstrated that motor imagery of tennis swings was enhanced when athletes physically held a racket, highlighting the contribution of somatosensory information to motor performance (11). Similarly, Yamashiro et al. (12) reported shorter somatosensory Go-P100 latency and reaction time in baseball players compared with track and field athletes, whereas auditory Go-N100 responses did not differ between them (12). These findings suggest that long-term sports training may preferentially induce modality-specific neural adaptations in the somatosensory system rather than generalized changes across sensory modalities. In addition, somatosensory evoked potentials (SEPs) have been proposed as a non-invasive neurophysiological method for assessing functional neuroplasticity within the sensorimotor system. Previous studies showed that SEP parameters, including both short- and long-latency components, are modulated by long-term training and are associated with behavioral indices such as reaction time and training history (13). Furthermore, SEP recordings provide a direct measure of neural activity with high temporal resolution, making them suitable for examining training-related neural adaptations in human movement contexts.

Importantly, physical fitness in adolescents involves coordination, balance, and rapid feedback, which are likely to depend on the efficiency of somatosensory processing even in non-athlete populations. However, to our knowledge, no study has directly examined the relationship between physical fitness components and somatosensory brain responses in adolescents. Investigating this relationship in non-athlete populations is critical for understanding the neural basis of fundamental motor development independent of sport-specific training.

Adolescence is a developmental period characterized by rapid physical, hormonal, and neural changes (14). Neuroimaging studies have shown that white matter volume increases through adolescence, while gray matter volume peaks in late childhood and declines during puberty (15, 16). In addition, boys and girls exhibit distinct developmental trajectories in physical fitness and motor performance, influenced by both hormonal and maturational factors (17, 18). Puberty-related increases in sex hormones also affect cortical development and sensorimotor network organization (19). These sex-stratified developmental pathways suggest that associations between somatosensory brain responses and physical fitness may differ between boys and girls during adolescence. Therefore, examining these relationships separately in boys and girls is important for accurately characterize the underlying neurophysiological mechanisms.

Based on this background, the present study aimed to examine the relationship between somatosensory brain responses and physical fitness in non-athlete adolescents, with particular attention to potential sex differences. We hypothesized that somatosensory brain responses would be associated with physical fitness components, reflecting developmental changes in the integration of sensory input and motor output during adolescence.

## 2 Materials and methods

### 2.1 Participants

Thirty-six pubertal children (sixteen boys and twenty girls) with right-handedness participated in this study. Handedness was assessed using the Edinburgh Handedness Inventory (20), and only children classified as right-handed were included. The age and anthropometric data of the participants are shown in Table 1. None of the participants had a history of neurological or psychiatric disorders. Informed consent was obtained from all participants and children's guardians. The procedures used complied with the Declaration of Helsinki regarding human experimentation. This study was approved by the Ethical Committee of Nara Women's University, Nara, Japan.

**Table 1:** Mean values for age and anthropometric and physical fitness data with SD.

Variable	Boys	Range for boys	Girls	Range for girls	Total	Total range	Cohen's d
Age (months)	175.9 (2.9)	172–181	174.9 (3.5)	169–181	175.4 (3.3)	169–181	0.31
Height (cm)	161.0 (9.1)*	141.0–178.3	155.4 (4.6)	148.8–165.0	157.5 (7.4)	141.0–178.3	0.77
Weight (kg)	48.9 (9.9)	27.0–64.4	45.1 (5.4)	34.5–60.0	46.5 (7.7)	27.0–64.4	0.48
50 M sprint (s)	8.1 (0.5)***	7.1–9.1	8.9 (0.6)	7.8–10.2	8.5 (0.7)	7.1–10.2	−1.45
Standing long jump (cm)	197.5 (24.3)**	152.0–230.0	176.3 (15.8)	139.0–200.0	185.4 (22.3)	139.0–230.0	1.04
Grip strength (kg)	29.0 (7.7)*	20.0–49.0	22.9 (2.9)	17.0–27.0	25.6 (6.3)	17.0–49.0	1.07
Sit-ups (*n*/30 s)	30.4 (6.6)**	23–46	24.6 (5.5)	15–34	27.1 (6.6)	15–46	0.96
Sit and reach (cm)	50.8 (10.0)	33.0–68.0	49.2 (8.0)	31.0–60.0	49.9 (8.9)	31.0–68.0	0.18
Side steps (*n*/20 s)	58.2 (7.0)**	43–70	52.0 (5.1)	46–67	54.6 (6.7)	43–70	1.02
Ball throw (m)	21.2 (4.8)***	12.0–30.0	12.3 (3.5)	6.0–19.0	16.4 (6.1)	6.0–30.0	2.12
Total score (points)	39.9 (8.3)	26–56	45.4 (8.2)	29–60	–	–	
Weekly exercise time (min)	753.4 (310.1)	165–1,300	734.5 (444.1)	130–1,980	742.9 (385.2)	130–1,980	0.05

Asterisks indicate significant differences between boys and girls.

* *p* < 0.05.

** *p* < 0.01.

*** *p* < 0.001.

### 2.2 Anthropometric and physical fitness tests

Data on anthropometric measurements and physical fitness tests were obtained from a part of analyses conducted annually, obtained in May 2022, at Nara Women's University Secondary School. The measurements were performed within a physical education class. Physical fitness tests were based on the guidelines recommended by the Ministry of Education, Culture, Sports, Science and Technology (MEXT) of Japan, which provide standardized and widely used measures for evaluating physical fitness in Japanese youth (21). Anthropometric and physical fitness tests for their age included: height, weight, 50 m sprint (speed of body movement), standing long jump (lower body explosive strength), grip strength (muscle strength), sit-ups (abdominal muscle strength/endurance), sit and reach (flexibility), side steps (agility), and ball throw (upper body explosive strength). The value for grip strength was calculated as the average grip strength for the left and right hands. Sit-ups were measured as the number of repetitions completed in 30 s. For the side steps, 3 parallel 200 cm long lines 100 cm apart were marked on the floor. The subjects were instructed to initially straddle the middle line and then touch the right line with their right foot. Then, they were asked to change direction and touch the left line with their left foot, still facing the front. This pattern was then repeated as fast as possible. The number of side steps completed in 20 s was recorded. The ball throw test consisted of a softball throw, and the longest distance achieved on two attempts was recorded. In the physical fitness tests, all students were encouraged to perform at their best. Before starting the physical fitness tests, standardized warm-up and stretching exercises were performed. Since the same physical fitness tests by MEXT were performed every year, the students had already experienced them. The total score was calculated in accordance with the standards set by MEXT. We also interviewed the students about their weekly exercise time. Weekly exercise time was assessed using the standardized questionnaire provided by MEXT of Japan. The questionnaire requires students to report the amount of time spent engaging in physical activity on each day of the week, and total weekly exercise time was calculated by summing the minutes reported for all seven days, as shown in the official MEXT format.

### 2.3 SEP recording procedure

EEG was recorded simultaneously with electric stimulation using Ag/AgCl disk electrodes placed on the scalp at Fz, Cz, Pz, and C3' (C3' was 2 cm posterior to C3), according to the International 10–20 System (Neuropack X1 system, Nihon-Kohden, Tokyo, Japan). C3' was selected to record somatosensory responses from the contralateral left primary somatosensory cortex (SI), where SEP components are typically maximal. Each scalp electrode was referenced to linked earlobes which were mathematically calculated as an averaged reference. In order to preclude interference noise due to eye movements or blinks exceeding 100 µV, an electrooculogram (EOG) was recorded bipolarly using a pair of electrodes placed 2 cm lateral to the lateral canthus of the right eye and 2 cm above the upper edge of the right orbit, and monitored online. During online acquisition, trials containing EOG or EEG signals exceeding 100 µV were automatically rejected by the EEG system. In addition, all remaining raw EEG data were carefully inspected offline on a trial-by-trial basis. Trials containing residual artifacts that did not exceed the 100 µV threshold but were clearly non-physiological in nature (e.g., unexplained electrical noise or muscle-related artifacts) were manually excluded from subsequent averaging. This two-step artifact rejection procedure (online automatic rejection followed by offline visual inspection) was applied consistently across all participants. Impedance was maintained at less than 5 kohm. All EEG signals were collected on a signal processor (Neuropack X1 system, Nihon-Kohden, Tokyo, Japan).

We used the standard constant current stimulator integrated in the Neuropack X1 system (Nihon-Kohden, Tokyo, Japan). This device can deliver stimulation and record EEG simultaneously. The electric stimulus was delivered to the right median nerve using a pair of felt-up electrodes. Stimulation was applied to the right side in all participants, who were all right-handed, to ensure methodological consistency and minimize variability related to hemispheric dominance. The stimulus duration was 0.2 ms, the stimulation frequency was 3 Hz, and the stimulus intensity was sufficient to produce a slight but definite twitch of the thumb (mean intensity: 4.2 ± 0.9 mA). This level of stimulation is not considered painful, and none of the participants reported discomfort during the SEP recording. Participants were asked to stay relaxed and not pay attention to the stimuli. Approximately two hundred stimuli were applied in total.

### 2.4 EEG data analysis

The bandpass filter was 1–1,500 Hz. The analysis window was 100 ms in total, consisting of a prestimulus baseline (−10 to 0 ms) and 90-ms poststimulus period, and the sampling rate was 5,000 Hz. A 60 Hz notch filter was applied to reduce line noise. The peak amplitude of each SEP component was measured using a peak-to-peak method. These recording and analysis settings were commonly used for SEP measurements, and implemented in accordance with the recommendations of the International Federation of Clinical Neurophysiology (22) and previous studies (23–26). The P12 component was not analyzed because it is the first component in the waveform and, therefore, has no preceding negative or positive peak from which a peak-to-peak amplitude can be calculated. Based on previous studies (23, 24), Fz and C3' were selected as the primary electrodes for analysis. Cz and Pz were included as supplementary recording sites to confirm the consistency of SEP waveforms across midline regions, but no statistical analyses were conducted using these electrodes. To ensure consistency with previous SEP studies using median nerve stimulation, we focused on short-latency components that are reliably identifiable and functionally meaningful. At Fz, we measured N15, P18, and N30, which reflect early fronto-sensorimotor processing. At C3', we measured N18, P22, N27, P45, and N60, which correspond to somatosensory cortical activity in contralateral somatosensory cortex and related pathways (23–26). To ensure the consistency and reliability of the peak amplitude measurements, all SEP components were independently evaluated by two or more trained researchers.

### 2.5 Statistical analysis

Firstly, we compared physical fitness data between boys and girls. This analysis was performed after checking that data had a normal distribution using the Kolmogorov–Smirnov test. If a normal distribution was confirmed, the data were subjected to one-way (sex) analysis of variance. If non-normal distribution was observed, the data underwent Mann–Whitney *U*-tests.

We analyzed bivariate correlations between the data of anthropometric and physical fitness tests (including the total score), weekly exercise time, and age in months and the amplitudes of each SEP component recorded at Fz and C3' electrodes. If a normal distribution was confirmed, Pearson's correlation was calculated. If non-parametric data were identified, Spearman's correlation was analyzed. Because a large number of bivariate correlations were performed, a total of 72 correlation analyses were conducted for each sex, we primarily considered correlations with *p* < 0.05 and an absolute r value greater than 0.50 as meaningful (27, 28). In addition, we applied Benjamini-Hochberg false discovery rate (FDR) correction (q = 0.20) as an exploratory control for multiple comparisons (29). In this procedure, *p*-values are ranked in ascending order and adjusted based on their rank and the total number of tests to obtain q-values, which represent the expected proportion of false discoveries among the rejected hypotheses. We reported the findings that remained below this threshold. In addition, to formally evaluate whether the associations between SEP amplitudes and physical fitness differed by sex, we performed multiple regression analyses, including the interaction of physical fitness components and sex within a general linear model framework. In these models, the physical fitness measure of interest was entered as the dependent variable. SEP amplitude and sex were entered as independent variables, and an interaction term between SEP amplitude and sex was included (SEP amplitude × sex). For interpretation of the magnitude of the observed associations, effect size indices were reported for the main analyses: *η*^2^ for ANOVA, correlation coefficients (r) for bivariate associations, and partial *η*^2^ for regression analyses. Significance was set at *p* < 0.05. An *a priori* power analysis was conducted using G*Power for correlation analyses. Assuming a large effect size (r = 0.50), an alpha level of 0.05, and a power of 0.80, the required sample size was estimated to be 29 participants.

## 3 Results

Table 1 presents the results obtained from anthropometric measurements, physical fitness tests, and weekly exercise time.

ANOVA showed significant differences between boys and girls in height [F (1, 34) = 6.653, *p* = 0.015, *η*^2^ = 0.240], 50 m sprint [F (1, 34) = 14.858, *p* < 0.001, *η*^2^ = 0.311], standing long jump [F (1, 34) = 9.823, *p* = 0.004, *η*^2^ = 0.229], grip strength (U = 86.000, Z = −2.365, *p* = 0.018), sit-ups [F (1, 34) = 7.967, *p* = 0.008, *η*^2^ = 0.194], side steps [F (1, 34) = 9.401, *p* = 0.004, *η*^2^ = 0.222], and ball throw [F (1, 34) = 39.680, *p* < 0.001, *η*^2^ = 0.546].

**Figure 1:**
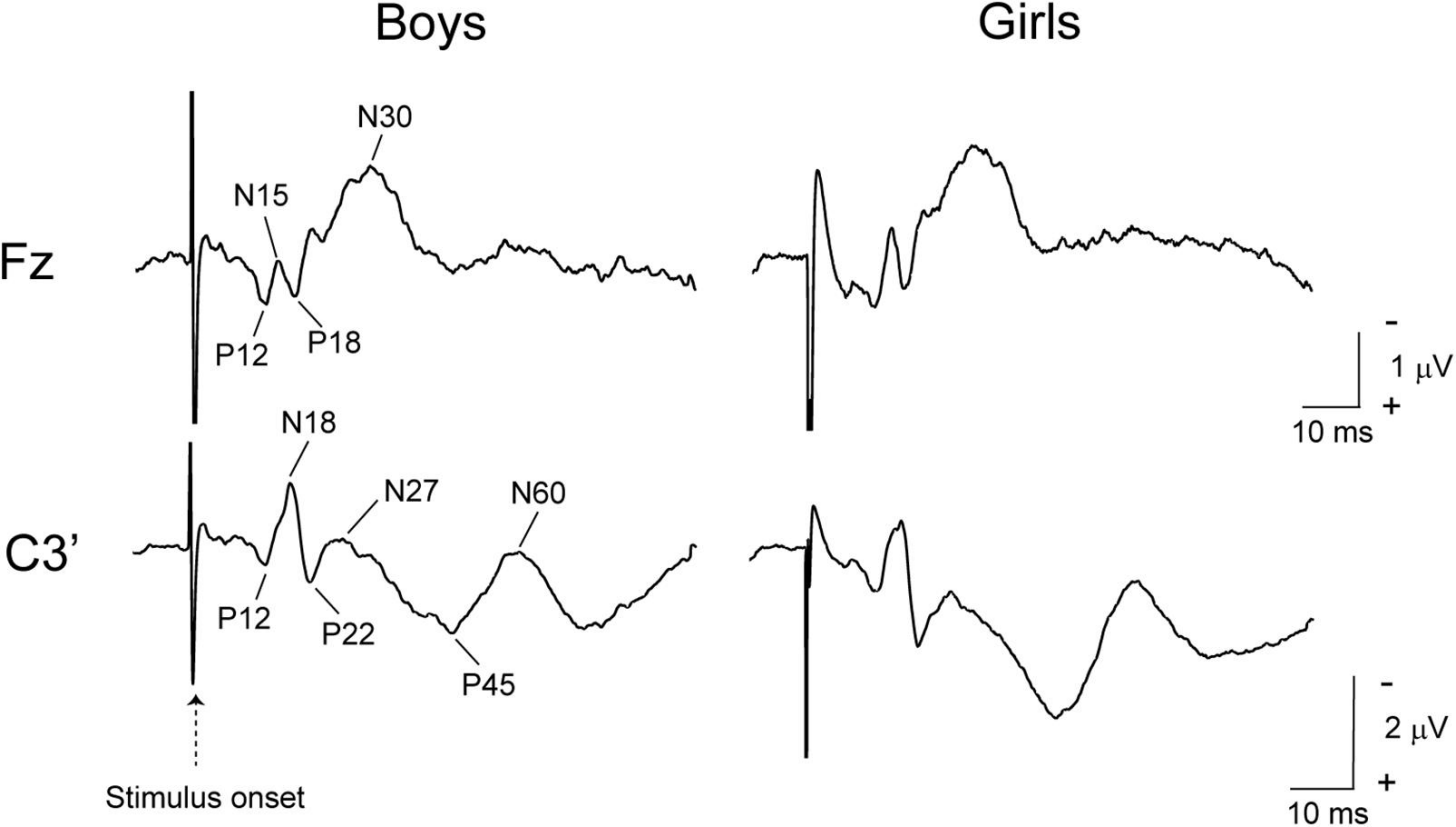
Grand-averaged SEP waveforms at Fz and C3’ across all participants.

Figure 1 presents grand-averaged SEP waveforms across all participants for each group at Fz and C3' electrodes. Table 2 shows average values for amplitudes of SEP at Fz and C3'.

**Table 2:** Mean values for peak amplitudes of each SEP component at Fz and C3’ with SD.

(µV)	Component	Boys	Girls	All
Fz	N15	1.2 (0.4)	1.6 (0.6)	1.4 (0.6)
	P18	1.2 (0.5)	1.4 (0.7)	1.3 (0.6)
	N30	2.6 (1.1)	2.6 (1.1)	2.6 (1.1)
C3'	N18	2.5 (1.5)	2.2 (1.2)	2.4 (1.3)
	P22	3.2 (1.6)	3.6 (1.9)	3.4 (1.8)
	N27	1.8 (1.1)	2.1 (1.5)	2.0 (1.3)
	P45	3.3 (1.7)	3.8 (1.8)	3.6 (1.7)
	N60	3.2 (1.0)	4.0 (2.3)	3.7 (1.9)

### 3.1 The relationship between physical fitness index and SEP components

Table 3 shows correlations between the data of physical fitness tests and peak amplitudes of SEP components.

**Table 3:** The r values of correlations between physical fitness data and peak amplitudes of SEP components.

Electrode	Component	Total score (points)	50 M sprint (s)	Standing long jump (cm)	Grip strength (kg)	Sit-ups (*n*/30 s)	Sit and reach (cm)	Side steps (*n*/20 s)	Ball throw (m)
Boys									
Fz	N15	0.322	−0.425	0.439	0.101	0.427	0.055	−0.320	0.268
	P18	−0.123	0.054	−0.190	−1.000	0.041	0.031	−0.185	−0.275
	N30	0.594*	−0.560*	0.537	0.465	0.657**	0.402	0.447	0.141
C3'	N18	0.199	−0.294	0.252	0.100	0.421	0.113	0.074	0.045
	P22	−0.024	−0.100	0.045	−0.110	0.193	0.007	−0.141	−0.075
	N27	−0.053	0.040	0.006	−0.266	0.035	−0.481	0.067	−0.147
	P45	−0.031	−0.159	0.162	−0.112	0.239	−0.306	−0.107	0.052
	N60	−0.305	0.194	−0.194	−0.315	0.054	−0.129	−0.439	−0.462
Girls									
Fz	N15	0.035	−0.064	−0.244	0.083	−0.151	−0.054	−0.007	0.345
	P18	0.171	−0.026	0.021	0.114	0.033	0.409	0.137	0.150
	N30	−0.268	0.383	−0.261	−0.031	−0.390	0.109	−0.296	−0.044
C3'	N18	−0.033	−0.245	−0.280	0.025	−0.115	0.072	−0.229	0.281
	P22	−0.058	0.012	−0.353	0.108	−0.222	−0.064	−0.122	0.100
	N27	−0.212	0.282	−0.585**	−0.119	−0.354	−0.198	−0.238	−0.136
	P45	−0.311	0.249	−0.499	−0.245	−0.253	0.004	−0.159	−0.194
	N60	−0.249	0.345	−0.491	−0.217	−0.211	0.073	−0.150	−0.344
All									
Fz	N15		0.027	0.004	−0.018	−0.052	0.094	−0.200	−0.061
	P18		0.061	−0.071	0.002	0.044	0.215	−0.036	−0.109
	N30		−0.300	0.179	0.130	0.159	0.262	0.111	0.042
C3'	N18		−0.282	0.036	0.154	0.184	0.121	0.036	0.328
	P22		0.036	−0.157	0.047	−0.065	−0.040	−0.154	−0.068
	N27		0.087	−0.322	−0.178	−0.118	−0.277	−0.112	−0.083
	P45		0.155	−0.248	−0.190	−0.135	−0.158	−0.190	−0.154
	N60		0.177	−0.407	−0.213	−0.070	0.086	−0.266	−0.265

Pubertal boys with higher total scores, faster times in the 50-m sprint, and greater number of sit-ups showed larger peak amplitudes. Pubertal girls with longer standing long jump distances showed smaller peak amplitudes.

* *p* < 0.05.

** *p* < 0.01.

**Figure 2:**
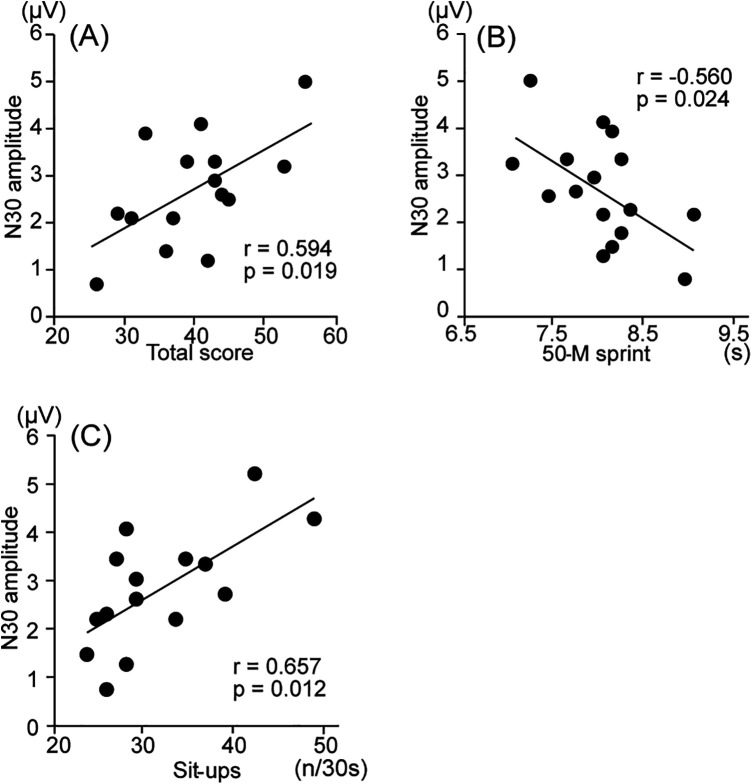
Correlation between physical fitness data and N30 amplitudes for boy participants. **(A)** The correlation between total score test and amplitude of N30 at Fz; **(B)** The correlation between the time in the 50 m sprint and amplitude of N30 at Fz; **(C)** The correlation between the number of sit-ups and amplitude of N30 at Fz.

**Figure 3:**
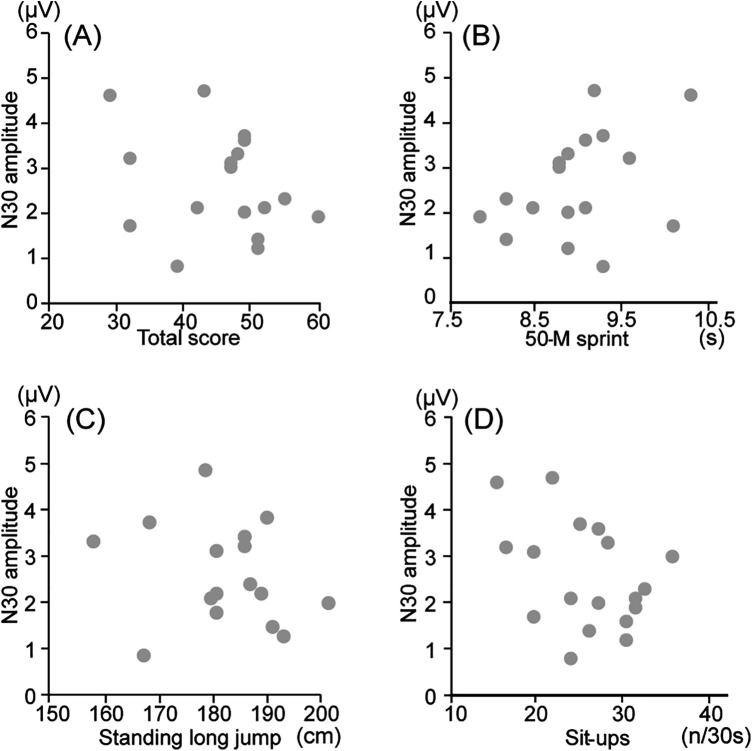
Correlation between physical fitness data and N30 amplitudes for girl participants. **(A)** The correlation between total score test and amplitude of N30 at Fz; **(B)** The correlation between the time in the 50 m sprint and amplitude of N30 at Fz; **(C)** The correlation between the distance achieved in the standing long jump and amplitude of N30 at Fz; **(D)** The correlation between the number of sit-ups and amplitude of N30 at Fz.

In boys, a significant positive correlation was observed between the total score in physical fitness tests and amplitude of N30 at Fz (*r* = 0.594, *p* = 0.019, *q* = 0.152), indicating that individuals with a higher total score showed a larger peak amplitude. A significant negative correlation was observed between the time for the 50-m sprint and amplitude of N30 at Fz (*r* = −0.560, *p* = 0.024, *q* = 0.192), indicating that individuals with faster times in the 50-m sprint showed larger peak amplitudes. A significant positive correlation was observed between the number of sit-ups and amplitude of N30 at Fz (*r* = 0.657, *p* = 0.008, *q* = 0.056), indicating that individuals who performed more sit-ups showed a larger peak amplitude (Figure 2). These correlations were not noted in girls (Figure 3). No significant correlations were observed between any record in physical fitness tests and the amplitudes of N15 and P18 at Fz, as well as N18, P22, N27, P45, and N60 at C3'.

**Figure 4:**
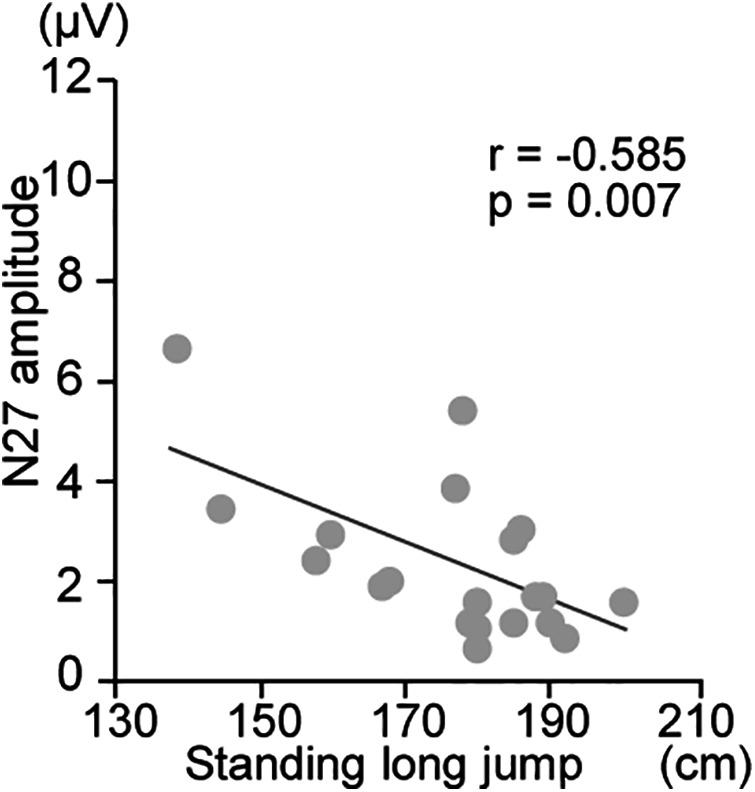
Correlation between physical fitness data and SEP amplitudes at C3’ for girl participants. The correlation between the distance achieved in the standing long jump and amplitude of N27 at C3’.

In girls, a significant negative correlation was observed between the distance in the standing long jump and the amplitude of N27 at C3' (*r* = −0.585, *p* = 0.007, *q* = 0.056), indicating that individuals with a longer standing long jump distance showed a smaller peak amplitude (Figure 4). No significant correlations were observed between the amplitudes of N15, P18, and N30 at Fz, as well as N18, P22, P45 and N60 at C3’ and any other records in physical fitness tests.

In the analysis including all participants, no significant correlations were observed in any other components at Fz and C3'.

We also performed multiple regression analyses within a general linear model framework for the variables that showed significant correlations to further clarify the main effects and interactions. These analyses revealed significant interactions between the amplitude of N30 at Fz and sex for the total physical fitness score (F = 6.572, *p* = 0.016, partial *η*^2^ = 0.190), 50 m sprint (F = 7.844, *p* = 0.009, partial *η*^2^ = 0.213), and sit-ups (F = 12.440, *p* = 0.001, partial *η*^2^ = 0.300). These results suggest that the associations between the amplitude of N30 at Fz and these physical fitness measures differ between boys and girls. In contrast, no significant interaction was observed between the amplitude of N27 at C3' and sex for the standing long jump (*F* = 1.832, *p* = 0.186, partial *η*^2^ = 0.059).

### 3.2 The relationship between weekly exercise time and SEP components

Table 4 shows the correlations between weekly exercise time and the peak amplitudes of SEP components. No significant correlations were observed for all variables.

**Table 4:** The r values of correlations between weekly exercise time and peak amplitudes of SEP components.

Electrode	Component	Boys	Girls	All
Fz	N15	0.208	−0.271	−0.107
	P18	0.041	−0.152	−0.003
	N30	0.345	−0.385	−0.074
C3'	N18	0.240	−0.215	0.038
	P22	0.168	−0.230	−0.017
	N27	−0.195	−0.185	−0.217
	P45	0.304	−0.274	−0.003
	N60	0.179	0.001	0.050

No significant correlations were observed for all variables.

## 4 Discussion

We investigated the relationship between physical fitness and somatosensory brain responses in pubertal children, by recording anthropometric and physical fitness tests and SEPs. The main findings were as follows. First, boys with higher physical fitness scores, particularly in sprinting and abdominal endurance, showed larger N30 amplitudes at Fz, suggesting enhanced sensorimotor integration. In contrast, girls did not exhibit the same pattern of associations, indicating potential sex-stratified differences in the neural mechanisms underlying physical fitness. Although these results suggest sex-related differences in the neural correlates of physical fitness during puberty, the mechanisms underlying these patterns are likely multifactorial and should be interpreted with caution.

There are two pathways of somatosensory processing in the human brain: a posterior pathway from Brodmann's area 3b of SI to areas 1 and 2; and an anterior pathway from areas 3b to 4, 6, and 8 (30). In SEPs, when the median nerve is stimulated, P12, N18, P22, N27, P45, and N60 components are recorded at centroparietal electrodes contralateral to the stimulated site, and P12, N15, P18, and N30 components are recorded at frontal electrodes (23, 24). These activities are distinguishable between electrodes, and the frontal activities are not a mirror image of centroparietal activities (31).

Among boys, individuals with higher total scores in physical fitness tests and better scores in the 50-m sprint and sit-ups showed a larger amplitude of N30 at Fz. The known generator sources of N30 at Fz are the prefrontal cortex (PFC), premotor cortex (PM), and primary motor cortex (MI), reflecting early sensorimotor integration and the engagement of motor planning and control (31, 32). Although these correlations do not suggest causality, they may indicate that boys with better motor performance exhibit more efficient neural processing related to movement initiation or planning. For example, the amplitude of N30 increased during observation of a complex finger movement, compared with the resting control (33). Nakata et al. (25) recorded the frontal N30 and long-latency N140, which was mainly generated from the secondary somatosensory cortex, during resting control and voluntary movement (25). They showed that the neural activities during voluntary movement affected the sources for N30 and vertex N140 in the same neuronal network simultaneously. Furthermore, MI receives inputs neuroanatomically from not only PM, but also SI (34). Taken together, the present findings may suggest that individual differences in N30 amplitude are related to variations in motor-related neural activity. However, interpretations regarding neural efficiency or specific sensorimotor integration mechanisms should be made with caution, as the present study does not directly assess these processes. These results may reflect an association between early frontal somatosensory processing and performance in tasks requiring speed, power, and core stability.

Girls with higher records in the standing long jump had smaller amplitudes of N27 at C3', respectively. N27 mainly reflects neural activity of Brodmann's area 2 (30, 35, 36). Interestingly, greater SEP amplitudes were observed in participants with poorer performance in the standing long jump. One possible explanation is that individuals with weaker motor output may rely more heavily on somatosensory input to compensate for deficits in movement execution. This interpretation is consistent with previous findings showing that tasks requiring greater somatosensory processing, such as manual object exploration (37) or ball rotation movements (38), are associated with increased SI activity. However, this explanation remains speculative. Other factors, such as differences in sensorimotor integration efficiency, task strategies, or neural development stages specific to girls during puberty, may also contribute. Moreover, while we hypothesize that better performance in the standing long jump may lead to more efficient and, thus, reduced somatosensory feedback processing, direct evidence supporting this mechanism in adolescent girls is limited. Taken together, these findings suggest a relationship between N27 amplitude and motor performance in girls, but the direction and mechanisms of this association remain to be clarified. Future studies incorporating additional physiological and behavioral measures, as well as larger sample sizes, are needed to better understand the neural mechanisms underlying this association.

Additionally, the sex-stratified differences observed in the relationship between physical fitness components and SEP amplitudes may also be influenced by maturational, hormonal, or sociocultural factors specific to puberty. In the regression analysis of the present study, a significant interaction between sex and the N30 amplitude at Fz was noted for the total physical fitness score, 50 m sprint, and sit-ups indicating that the association between somatosensory processing and global motor competence differed between boys and girls. This sex-stratified pattern suggests that the neural mechanisms linking somatosensory processing with motor performance may rely on developmental processes that manifest differently across sexes during puberty. For example, previous neuroimaging studies showed that cortical maturation tends to occur earlier in girls than boys, potentially affecting the timing and efficiency of somatosensory processing (39). Hormonal changes during puberty, such as increased estrogen levels in girls, may also modulate sensory processing and contribute to differences in neural activation patterns (40). Furthermore, sociocultural differences in physical activity engagement, such as variations in sport participation or movement experiences between boys and girls, may affect use-dependent plasticity in somatosensory processing (41). These factors, alone or in combination, may help explain the greater SEP amplitudes observed in girls with poorer motor performance. Future studies incorporating neuroendocrine markers, pubertal staging, and detailed assessments of physical activity type may help clarify these complex influences.

We also hypothesized that physical fitness was influenced by the muscle power of the participants (42, 43). That is, if significant correlations between physical fitness components and amplitudes of SEP components were found when analyzing data from all participants, these relationships could be attributed to muscle power rather than sex differences. To clarify this, we conducted an additional analysis examining the relationship between physical fitness components and amplitudes of SEP components across all the participants, including all the boys and girls. The results revealed no significant correlations between physical fitness components and the amplitudes of any SEP components at Fz or C3'. These findings suggest that the observed relationship between physical fitness and somatosensory brain responses are more likely influenced by sex differences (39, 40) rather than muscle power.

To elucidate the relationship between daily physical activity and somatosensory processing, we examined the weekly exercise time and the amplitudes of SEP components. However, no significant correlations were observed for any of the variables (Table 4), suggesting that daily physical activity does not contribute to somatosensory processing. Kidokoro et al. (44) investigated the relationship between scores in physical fitness tests and daily physical activity levels among 145 pubertal boys and 155 pubertal girls (junior high school students) (44). They showed a significant positive correlation in pubertal girls, indicating that pubertal girls with higher scores in physical fitness tests had higher levels of physical activity. In contrast, our findings revealed that somatosensory brain responses were associated with physical fitness. However, weekly exercise time was assessed using a self-reported questionnaire, which may have limited accuracy, particularly in adolescent populations. In addition, the measure did not account for factors such as activity intensity, type of physical activity, or sport specialization. Therefore, the absence of associations between physical activity and SEP measures should be interpreted with caution.

The present study had several limitations. First, we focused on the relationship only in pubertal children between physical fitness and somatosensory brain responses. Although participants were classified as adolescents based on school grade and age range, pubertal status was not directly assessed (e.g., Tanner stage or hormonal markers), and individual differences in pubertal development may have influenced the observed associations. Therefore, the present findings should be interpreted with caution. Future studies including prepubertal children as well as young and older adults are needed to determine whether the observed relationships and sex-related differences are specific to adolescence. In addition, increasing the sample size and stratifying participants based on physical fitness levels may provide a more detailed understanding of these associations. Second, the relatively small sample size in relation to the number of statistical tests performed represents an additional limitation. Because correlations were examined between multiple SEP components and physical fitness indicators, the statistical power may have been limited, and the stability of the observed correlations cannot be fully ensured. Although false discovery rate correction was applied, the possibility of false-positive findings cannot be excluded. Therefore, the present results should be considered exploratory, and future studies with larger samples are required to confirm these findings. In addition, weekly physical activity was assessed using self-reported data rather than objective measures such as accelerometry, which may have introduced measurement bias. Third, the present study mainly focused on “short-latency” SEPs to evaluate somatosensory processing. By recording “long-latency” SEPs and/or event-related potentials (ERPs) with somatosensory stimuli, it is possible to clarify more detailed neural mechanisms and assess higher somatosensory cognitive function.

## 5 Conclusions

The present study examined the relationship between physical fitness and somatosensory brain responses during puberty. Among boys, higher physical fitness, particularly in sprinting and abdominal endurance, was associated with larger N30 amplitudes at Fz, suggesting a link between motor performance and early frontal somatosensory processing. In contrast, among girls, better performance in the standing long jump was associated with smaller N27 amplitudes at C3', indicating a different pattern of association in centroparietal somatosensory processing. These findings indicate sex-specific associations between physical fitness and somatosensory brain responses during adolescence. However, given the exploratory nature of the study and the potential influence of developmental and methodological factors, these results should be interpreted with caution and require confirmation in future studies.

## Data Availability

The raw data supporting the conclusions of this article will be made available by the authors, without undue reservation.
